# WT-1, BAALC, and ERG Expressions in Iranian Patients with Acute Myeloid Leukemia Pre- and Post-chemotherapy

**DOI:** 10.34172/apb.2021.021

**Published:** 2020-11-07

**Authors:** Hossein Mehralizadeh, Mohammad Reza Aliparasti, Mehdi Talebi, Shabnam Salekzamani, Shohreh Almasi, Morteza Raeisi, Mehdi Yousefi, AliAkbar Movassaghpour

**Affiliations:** ^1^Stem Cell Research Center, Tabriz University of Medical Sciences, Tabriz, Iran.; ^2^Immunology Research Center, Tabriz University of Medical Sciences, Tabriz, Iran.; ^3^Department of Applied Cell Science, School of Advance Medical Sciences, Tabriz University of Medical Sciences, Tabriz, Iran.; ^4^Department of Nutrition, School of Public Health, Bushehr University of Medical Sciences, Bushehr, Iran.; ^5^Hematology and Oncology Research Center, Tabriz University of Medical Sciences, Tabriz, Iran.

**Keywords:** Acute myeloid leukemia, WT-1, BAALC, ERG, Gene expression, Chemotherapy

## Abstract

***Purpose:*** Acute myeloid leukemia (AML) is the most prevalent acute leukemia in adults. It possesses different cytogenetic and molecular features. The expression of Wilms tumor-1 *(WT1)*, brain and acute leukemia, cytoplasmic *(BAALC)* and ETS-related gene *(ERG)* might be considered as prognostic factors in AML patients. The aim of this study was to determine the mRNA expressions of *WT-1*, *BAALC* and *ERG* genes in bone marrow of mononuclear cells and their effects on complete remission in the Iranian AML patients, pre- and post- chemotherapy.

***Methods:*** Forty AML patients with normal karyotype were evaluated. The mRNA gene expressions were measured with quantitative real-time PCR in bone marrow of mononuclear cells of AML patients at the baseline and after chemotherapy. The subtypes of AML and flow cytometry panel were also assessed. Complete remission (CR) after the treatment was addressed for all patients.

***Results:*** The mRNA expressions of *WT-1, BAALC* and *ERG* were significantly decreased after the treatment (*p* = 0.001, 0.017, 0.036). *WT-1* mRNA expression was inversely correlated with CR after chemotherapy (*P* =0.024). There was also significant correlation between baseline expression of BAALC and CR (*P* =0.046). No significant correlation was observed between ERG and CR pre- and post- chemotherapy (*P* =0.464 and 0.781). There was also significant correlation between *BAALC* mRNA expression and CD34+ (*P* <0.001).

***Conclusion:*** The present study showed that* WT-1* decreased significantly after standard chemotherapy which could have favorable effects on CR. Also, the high expression of *BAALC* could have a poor prognostic role in AML patients. The identification of these gene expressions can be an efficient approach in targeted therapy among AML patients.

## Introduction

Acute myeloid leukemia (AML) is the most prevalent acute leukemia in adults encompassing 80% of cases of this group.^1^ It is a malignancy characterized by the abnormal differentiation and proliferation of myeloid stem cells clonal population.^2^ Some special chromosomal alterations can lead to the formation of chimeric proteins which in turn may increase the likelihood of malignant transformation.^3^

AML is a heterogeneous disease with possessing different cytogenetic and molecular features which are considered as the key prognostic factors predicting the risk of survival and relapse.^3^ Recent advances in treatment protocols are based on these prognostic factors contributing to individualized therapy and risk-adapted intensification.^4^

The Wilms tumor 1 (*WT-1*) locus in chromosomal region 11p13; encodes a transcription factor highly expressed in the cells of the majority of leukemia patients at diagnosis, and apparently participates in leukemogenesis.^5^ High expression of *WT-1* in acute leukemia has been reported to represent a molecular marker of malignant hematopoiesis and was associated with fewer remissions and poor overall survival.^6,7^

Another molecular change in AML is the deregulated expression of brain and acute leukemia-cytoplasmic (*BAALC*) gene. *BAALC,* which maps on chromosome 8 at 8q22.3, is involved in neuroectodermal and hematopoietic development.^8^ The expression of*BAALC* was reported as a precursor for hematopoiesis like the cluster of differentiation CD34+ in early hematopoietic cells.^9^ High expression of that was found in bone marrow of AML patients with a normal karyotype^10^ and identified as anindependent poor prognostic indicator, associated with shorter survival.^11^

No specific role was explained for *BAALC* in leukemogenesis, but it was suggested that it blocks myeloid differentiation.^12^ It was suggested that high expression of BAALC is associated with a poor outcome in AML patients.^13^ Moreover, ETS-related gene (*ERG*) located on chromosome band 21q22, is a transcription factor required for normal hematopoiesis.^14^ The elevated expression of *ERG* was demonstrated in cytogenetically normal AML patients and has a negative role in the prognosis of AML.^15,16^ The role of standard treatment in the expression of these genes as well as the effect of post- treatment molecular changes on complete remission (CR) are not clearly investigated in the Iranian population.

In a study in North-East of Iran, the overexpression of *BAALC* was observed in AML patients aged 2 to 77. The up-regulation of *BAALC* was reported with survivability reduction.^17^ In another study, high level of *BAALC* in pediatric AML patients was associated with lower overall survival rate.^18^ High expression of *WT-1* was also shown in a study on Iranian AML patients.^19^ However, no study was conducted to evaluate these genes expression in AML at pre- and post-treatment phases.

Therefore, this study was designed to assess the expression of *WT-1*, *BAALC*, and *ERG* in normal karyotype of *de novo* AML patients and to compare the impact of standard chemotherapy on the expressions of these genes.

## Materials and Methods

### Patients

The study was performed on 40 normal karyotype de novo AML patients referred to Shahid Ghazi Tabatabai hospital in Tabriz, the Oncology and Hematology center in North-West of Iran, throughout the period between October 2018 and October 2019. The diagnosis of AML was confirmed by morphology and flow cytometry of bone marrow samples. Cytogenetic screening was also performed to select the normal karyotype patients. Patients’ karyotype was analyzed based on bone marrow of leukemic cells metaphases. Furthermore, AML subtypes were addressed based on French–American–British classification. The patients aged > 18 with diagnosis of AML were included in the study. Patients with other types of cancer or with secondary AML were excluded. All patients were undergone standard induction therapy according to 7-3 protocol (7 days with Cytarabine and 3 days with Daunorubicin or idarubicin). The second bone marrow sampling was done 2 weeks after the last chemotherapy. CR was assessed based on blast count less than 5%.

## Laboratory analysis

### Flow cytometry analysis 

Bone marrow samples were collected at diagnosis. The volume of bone marrow samples were 5 to 10 ml and collected on sterile ethylenediaminetetraacetic acid (EDTA) tube. For immunophenotyping analysis BD FACS Calibur (Becton, Dickinson Fluorescence Activated Cell Sorter (Becton, Dickinson Company, CA, USA) was used. The CD markers for Acute Leukemia Panel were: TdT, cy MPO, HLA-DR, GLYCO A, CD2, CD3, CD7, CD10, CD11b, CD13, CD14, CD15, CD19, CD20, CD22, CD33, CD34, CD38, CD41, CD45, CD61, CD117.

### RNA extraction and cDNA synthesis 

First, mononuclear cells were centrifuged by Ficoll-Hypaque density gradient. Total RNA extraction from mononuclear cells and blasts was done using Trizol Reagent (Invitrogen, USA) according to the manufacturer’s instructions. Total RNA (1 μg) was used for cDNAs synthesis with RevertAid™ H Minus Reverse Transcriptase (RT) (200U), primers (10 μM), dNTPs (1 mM), and RiboLock RNase inhibitor (20 U). The process was conducted at diagnosis and after chemotherapy.

### Quantitative real-time PCR

The gene expressions of *WT-1*, *BAALC*, and *ERG* were analyzed by quantitative Real-Time PCR at diagnosis and after chemotherapy by Life Science System (Rotor-Gene 6000). cDNA was diluted by 4-fold, then 2 μL was used in each PCR reaction with the volume of 15μL. It contained 150 nM of primers and 1X FastStart SYBR Green Master (Roche). Sequences of primers are listed in Table 1. The expression levels of *β-Actin* (ACTB), as a reference gene, were used to calculate relative expression levels. Data were shown as a ratio of the target gene/ACTB. The relative quantification (RQ) was performed by 2^-ΔΔCt)^: expression of target genes / *β* -*actin* Treated / Untreated = (1+E) ^-Ct target gene^ / (1+E) ^-Ct β-actin^ Treated / (1+E) ^-Ct target gene^ / (1+E) ^-Ct β-actin^ Untreated.

**Table 1:** Sequences of primers

***WT-1***	Forward	5’-GATAACCACACAACGCCCATC-3’
	Reverse	5’-CACACGTCGCACATCCTGAAT-3’
***BAALC***	Forward	5’-GCCCTCTGACCCAGAAACAG-3’
	Reverse	5’-CTTTTGCAGGCATTCTCTTAGCA-3’
***ERG***	Forward	5’-AACGAGCGCAGAGTTATCGT-3’
	Reverse	5’-GTGAGCCTCTGGAAGTCGTC-3’
***β*** ***-Actin***	Forward	5’-GCTGTGCTACGTCGCCCTG-3’
	Reverse	5’-GGAGGAGCTGGAAGCAGCC-3’

### Standard curve

A standard curve was used to determine the efficiency of RT-PCR reactions. To perform the curve, a positive PCR product was diluted serially by 10-fold. The logarithmic of concentrations of customary RT-PCR were plotted against the target gene cycling threshold (Ct) of serial dilution. The efficiencies of *WT-1*, *BAALC*, *ERG* and *β-Actin* were 96%, 93%, 97% and 96%, respectively.

### Statistical analysis 

For statistical analyses the SPSS software (ver. 21; IBM Corp., Armonk, NY, USA) was used; the significance of *p-values was considered less than* 0.05. Normality of data distribution was checked based on descriptive status. Normal-distributed data were expressed as mean ± SD, and non-parametric data were shown as median (75%, 25%). For possible difference in gene expression pre- and post-chemotherapy, Wilcoxon-signed rank test was utilized. The association between gene expression and other variables were performed using Spearman correlation-coefficient test. The differences between non-parametric variables were checked by chi-square test.

## Results

### Patients 

In the present study, 40 de novo AML patients were studied. The mean ± SD age of the patients was 36.75 ± 13.38 years. Fifty percent of patients were male. Patients’ clinical characteristics are shown in Table 2. White blood cell count ranged from 570 to 13.5×10^3^ with the median of 139×10^3^. The minimum and maximum blast count was 153 and 9×10,respectively. The flow cytometry analysis showed that the most and the least patients were in M2 and M3 subtypes with 40% and 10%, respectively. Thirty out of 40 patients were found to be leukemic CD34+ positive.

**Table 2:** Basic characteristics of patients

**Characteristics**	**Median (min, max)**
Age	36.75 ± 13.38*
Sex (male)	20 (50%)
WBC count/L	135×10^3^(570-139×10^3^)
Blast count /L	3525.50 (153.00- 90880.00)
Blast (%)	24.50 (7.00-71.00)
Platelet count	63 × 10^3^(13 ×10^3^ – 575 ×10^3^)
RBC	3.06 (2.27- 4.58)
HB	8.7 (6.8- 11.20)
MCV	92.75 (80.00 – 106.00)
Neutrophil	2.48 ×10^3^(210- 58.9 ×10^3^)
Lymphocyte	4.22 ×10^3^ (200 – 27.4 ×10^3^)
FAB classification	
M1	6 (15%)
M2	16 (40%)
M3	4 (10%)
M4	8 (20%)
M5	6 (15%)
CD34	
Positive	(30)75%
Negative	(10) 25%

* Mean ± SD.

### Gene expression 

#### Comparison of WT-1, BAALC and ERG gene expressions in AML patients in pre- and post- chemotherapy phases

**Figure 1:**
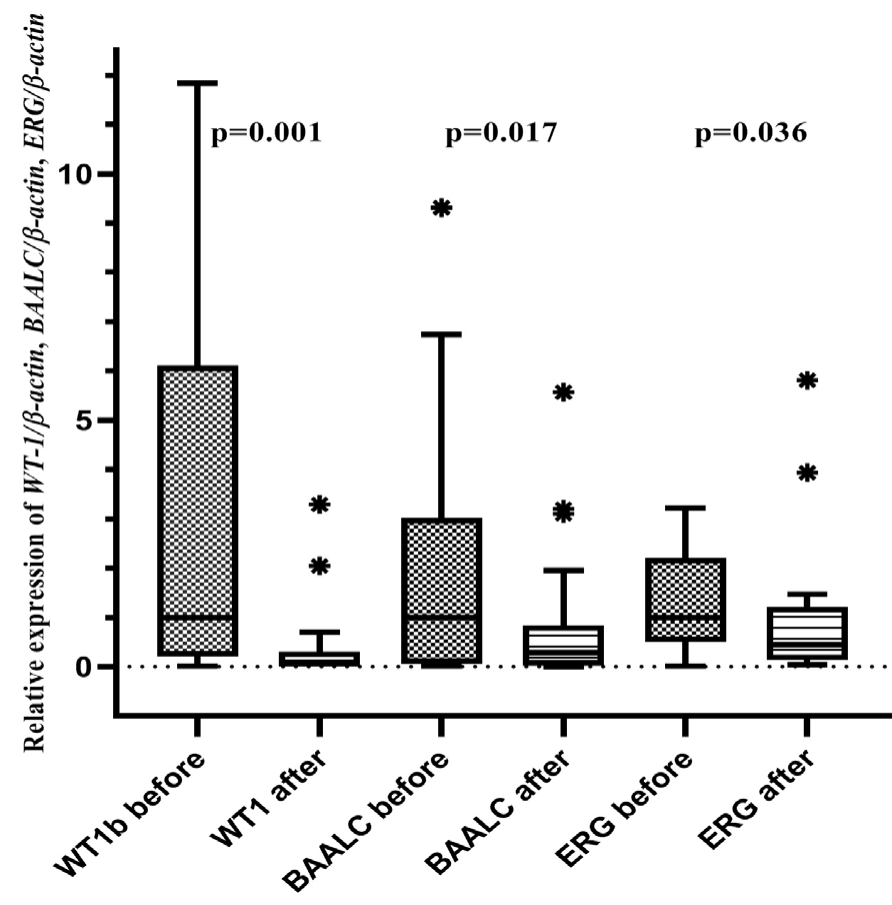
*WT-1*, *BAALC* and *ERG* expressions in BM of AML patients in pre- and post-chemotherapy phases.

The gene expression of *WT-1*, *BAALC* and *ERG* decreased significantly in the post-chemotherapy phase. The median of *WT-1*, *BAALC* and *ERG* pre- and post- chemotherapy is shown in Figure 1.

#### Correlation between WT-1, BAALC and ERG with CR

There was an inverse significant correlation between post-chemotherapy expression of *WT-1* and CR (r= -0.387, *P* =0.024). Moreover, the expression of *BAALC* was correlated inversely with CR before chemotherapy (r= -0.318, *P* = 0.046). There was no significant correlation between *ERG* and CR pre- and post-chemotherapy (*P* =0.464 and 0.781) (Table 3).

**Table 3:** Correlation between *WT-1*, *BAALC* and *ERG* expressions and complete remission pre- and post-chemotherapy

	**Complete Remission**			
	**Pre- chemotherapy**		**Post- chemotherapy**	
	**r**	***P***	**r**	***P***
*WT-1*	0.252	0.138	-0.387	**0.024**
*BAALC*	-0.318	**0.046**	0.027	0.861
*ERG*	0.118	0.464	-0.045	0.781

#### Gene expressions in subtypes

The expressions of *WT-1*, *BAALC* and *ERG* were categorized in the form of high (>median) and low (≤ median). Table 4 depicts the FAB classification in relation to low and high expression for *WT-1*, *BAALC* and *ERG*. The results showed that, all patients in subtype M3 had high expression of *WT-1*. Also, in subtype M2, 67% of the patients showed high expression of *WT-1*. However, there were no significant differences among subtypes of AML in terms of *WT-1* expression (*P* =0.08).

**Table 4:** Comparison of *WT-1*, *BAALC* and *ERG* expression among AML subtypes and CD34

	***WT-1*** **expression**		***P*** *	***BAALC*** **expression**		***P*** *	***ERG*** **expression**		***P*** *
	**High expression**	**Low expression**		**High expression**	**Low expression**		**High expression**	**Low expression**	
FAB classification			0.080			0.412			0.411
M1	2 (33.3%)	4 (66.7%)		2 (33.3%)	4 (66.7%)		4 (66.7%)	2 (33.3%)	
M2	8 (66.7%)	4 (33.3%)		10 (62.5%)	6 (37.5%)		10 (62.5%)	6 (37.5%)	
M3	4 (100%)	0 (0%)		2 (50.0%)	2 (50.0%)		2 (50.0%)	2 (50.0%)	
M4	2 (25.0)	6 (75.0%)		2 (25.0%)	6 (75.0%)		2 (25.0%)	6 (75.0%)	
M5	2 (33.3%)	4 (66.7%)		4 (66.7%)	2 (33.3%)		2 (33.3%)	4 (66.7%)	
CD34			0.457			<0.001			0.028
Positive	14 (77.8%)	12 (66.7%)		20 (100%)	10 (50.0%)		18 (90.0%)	12 (60.0%)	
Negative	4 (22.2%)	6 (33.3%)		0 (0%)	10 (50%)		2 (10.0%)	8 (40.0%)	

*Chi-square test.

Regarding *BAALC*, 66.7% of subtype M5 were high expressers. In subtypes M2, the percentages of high and low expressions were similar. For *ERG*, 66.7% and 62.5% of subtype M1 and M2 were high expressers. There were no significant differences between AML subtypes in terms of *ERG* and *BAALC* expressions (*P* >0.05) (Table 4).

#### Comparison of WT-1, BAALC and ERG expression in relation to CD34+

Table 4 represents the differences of *WT-1*, *BAALC* and *ERG* expressions in AML patients with CD34 positive (CD34^+^) in comparison with CD34 negative (CD34-). There were no significant differences in *WT-1* expressions between two groups. *BAALC* and *ERG* expressions were significantly higher in CD34^+^ AML patients (*P* <0.001 and 0.028, respectively).

#### Correlation between WT-1, BAALC and ERG expression and hematologic characteristics

The results showed that (Table 5),*WT-1* expression had inverse and significant correlations with platelet and neutrophil counts (r= -0.349, *P* =0.037; r= -0.351, *P* =0.036, respectively). There was also a significant correlation between *BAALC* expression and blast count at baseline (r=0.337, *P* =0.034). Furthermore, *ERG* expression was inversely correlated with platelet count (r= -0.581, *P* <0.001).

**Table 5:** Correlation between *WT-1*, *BAALC* and *ERG* expression and hematologic characteristics

	**Platelet**		**WBC**		**Blast count**		**Neutrophil**	
	**r**	***P***	**r**	***P***	**r**	***P***	**r**	***P***
*WT-1*	-0.349	0.037	0.096	0.578	0.009	0.957	-0.351	0.036
*BAALC*	-0.189	0.249	0.291	0.068	0.337	0.034	0.253	0.115
*ERG*	-0.581	<0.001	0.066	0.687	0.167	0.304	-0.170	0.295

## Discussion

In the present study, 35% of the patients reached CR at post-chemotherapy phase. *WT-1*, *BAALC* and *ERG* gene expressions in bone marrow decreased significantly after chemotherapy. The most reduction was observed in *WT-1* expression from 1.009 to 0.084. Furthermore, there was an inverse association between the expression of *WT-1* post- chemotherapy and CR. Parallel to our study, Ujj et al^16^ reported that CR was achieved in patients whose *WT-1* gene expression changed from positive to negative during therapy. They conclude that the affected level of *WT-1* expression after therapy could be a prognostic factor in AML patients. In line with that, previous studies reported that the ratio of *WT-1* in pre- and post- chemotherapy phases was considered as a predictor of disease outcome in AML patients who have undergone “3+7” chemotherapy.^20^ Moreover, in a study by Anderson et al it was shown that a 1 log reduction in *WT-1* expression in one month of chemotherapy was significantly correlated with the overall survival; and the level of *WT-1* in diagnosis was not found to be a prognostic marker in these patients.^4^ These results showed that early and deep reduction of *WT-1* expression can be used as a useful marker for disease outcome in AML patients. The role of *WT-1* in hematologic malignancies has not been clearly understood. Earlier studies have reported the involvement of *WT-1* not only in proliferation but also in the inhibition of apoptosis in tumor cell cultures.^21^ Our findings showed that the standard chemotherapy was partially successful in *WT-1* reduction that was correlated with CR. Opposite to *WT-1*, our results depicted that *BAALC* expression at diagnosis was inversely correlated with CR. The expression of *BAALC* decreased significantly after chemotherapy, but the reduction was not as high as the reduction in *WT-1*. No significant correlation between post- chemotherapy *BAALC* expression and CR was found. Former studies also found that high *BAALC* expression at diagnosis had negative prognostic effect in AML patients.^22-24^, In a study by Soliman, et al^9^ there was no difference in *BAALC* expression pre- and post- chemotherapy which was in contrast of ours. However, in line with our findings, high *BAALC* expression was significantly correlated with lower CR.^9^ Baldus et al^25^ suggested that the overexpression of *BAALC* could determine patients’ resistance to treatment. However, in AML patients who have undergone allogeneic hematopoietic stem cell transplantation, *BAALC* expression had no prognostic effect.^26^ *BAALC* was found to block myeloid differentiation and promote leukemogenesis.^12^ Furthermore, in pediatric AML patients, BAALC and ERG expressions were shown to associate with low induction remission.^27,28^ BAALC overexpression was found in a group of genes which were identified as a high risk group for survival with MN1, SPARC, HOPX genes.^22^

In our study, the levels of *ERG* decreased after treatment. There was no significant correlation between *ERG* expression and CR pre- and post- chemotherapy phases. In contrast, high *ERG* was associated with poor CR in the Soliman study.^9^ Also, Marcucci et al, in a 5.7 year follow-up study, concluded that *ERG* expression could be an independent prognostic factor in AML patients.^15^ In a study of 50 AML patients, CR was different between low and high expression of ERG.^29^ Moreover, in a meta-analysis of seven studies, it was showed that high *ERG* expression was associated with lower CR and higher relapse in cytogenetically normal AML patients.^14^ *ERG* was involved in cell differentiation, proliferation and apoptosis. It also plays a role in leukemogenesis as a fusion partner with the *FUS* gene in recurrent AML patients.^30,31^

Our analysis showed that all patients in subtype M3 had high expression of *WT-1* at diagnosis and the least percentage of the patents in *WT-1* expression were M1 and M5. Accordingly, in a study of forty-three adult AML patients, the highest and the lowest expressions of *WT-1* were found in M3 and M5, respectively.^4^ However, similar to our study, no significant difference was found in *WT-1* expressions among AML subtypes.^4^ In contrast, in a study in Mashhad, Iran, the highest percentage of *WT-1* expression was shown in subtype M1 and the lowest was in M6.^19^ In our study, there was no patient in M6 subtype.

Highest expression of *BAALC* and *ERG* were observed in M5 and M1, respectively. There were also no significant differences among subtypes in terms of *BAALC* and *ERG* expressions at diagnosis. In line with our result, in an Egyptian study, the differences in *BAALC* and *ERG* expressions among AML subtypes were not significant.^9^ Also, in this study, the number of patients in M1 was the highest in terms of *BAALC* expression and in M2 and M3 in terms of *ERG* expression^9^ which was different from our study. In a study of Zhang et al,^26^ the M0 subtype had the highest expressions of *BAALC* and *ERG* in AML patients and the lowest *BAALC* expression was in M5 that was opposite to our study groups.

We also compared the *WT-1*, *BAALC*, and *ERG* expressions in terms of CD34+. The findings showed that only *BAALC* had significantly higher expression in CD34+ patients which was similar to a study of Damiani et al^8^ on 175 AML patients in Italy. In another study the expression of BAALC was higher in CD34+ patients with AML.^32^ However, some studies found no significant difference in CD34+ between high and low expression groups of BAALC.^29^ There was also no significant difference between CD34^+^ and CD34- in terms of *WT-1* expression in Anderson et al study^4^ that was similar to our results.

Furthermore, *WT-1* expression had significant correlations with platelet and neutrophil counts. Also, *BAALC* was significantly correlated with blast count. In a study by Zhou et al,^33^ a substantial amount of bone marrow blast counts were observed in AML patients who highly expressed *BAALC* compared to low-expressed ones. Also, high expression of *BAALC* was associated with increased blasts in Chinese AML patients.^34^ In contrast, a number of former studies found no significant correlation between *BAALC* expression and blast count.^9,20,22^

This study had some strengths and limitations. This is the first study that was conducted in North-West of Iran and the patients were selected from the referral hospital to which patients with diverse ethnics referred. However, due to limited financial support, we were unable to evaluate the mutations that are molecular abnormalities in AML patients. Also, the small number of patients was another limitation for our study. Future studies with large sample size and longer duration are warranted.

## Conclusion

In conclusion, we found a significant decrease in *WT-1*, *BAALC*, and *ERG* expressions after chemotherapy; the decline in *WT-1* expression was significantly correlated with CR. Also, *BAALC* overexpression at diagnosis could be a prognostic marker in AML patients.

## Ethical Issue

The study was approved by Committee of Ethics of Tabriz University of Medical Sciences (IR.TBZMED.REC.1398.897). Written informed consent was obtained from all patients. Written informed consent was obtained from all patients.

## Conflict of Interest

The authors declare no conflict of interest.

## Acknowledgments

This work has been done as part of the M.Sc. thesis for Hossein Mehralizadeh. This study was supported by Stem Cell Research Center at Tabriz University of Medical Sciences, Iran [NO. 63168]. Authors would like to acknowledge Shahid Ghazi Tabatabai hospital, Stem Cell Research Center, and Department of Immunology at Tabriz University of Medical Sciences (Iran) for their great help and cooperation. We also appreciate all the patients who participated in this study.
